# Pasting and Rheological Properties of Starch Paste/Gels in a Sugar-Acid System

**DOI:** 10.3390/foods11244060

**Published:** 2022-12-15

**Authors:** Ployfon Boonkor, Leonard M. C. Sagis, Namfone Lumdubwong

**Affiliations:** 1 Department of Food Science and Technology, Faculty of Agro-Industry, Kasetsart University, Bangkok 10900, Thailand; 2 Physics and Physical Chemistry of Food, Department of Agrotechnology and Food Sciences, Wageningen University & Research, Bornse Weilanden 9, 6708 WG Wageningen, The Netherlands

**Keywords:** granule size, amylose content, pasting properties, sugar–acid system, small amplitude oscillatory shear (SAOS), large amplitude oscillatory shear (LAOS), nonlinear rheology, Lissajous plot

## Abstract

This study was to investigate the impact of granule size, amylose content, and starch molecular characteristics on pasting and rheological properties of starch paste/gels in neutral (water) and sugar–acid systems. Normal rice starch (RS), waxy rice starch (WRS), normal tapioca starch (TS), and waxy tapioca starch (WTS) representing small-granule starches and intermediate-granule starches respectively, were used in the study. Impacts of granule size, AM content, and their synergistic effects resulted in different starch susceptibility to acid hydrolysis and interactions between starch and sucrose-water, yielding different paste viscosities in both systems. The high molecular weight ($\bar{M_{w}}$) and linearity of amylopectin and amylose molecules increased the consistency of starch pastes. RS produced a stronger and more brittle gel than other starch gels in both neutral and sugar–acid systems. The results indicated the impact of the effect of granule size and amylose content on starch gel behaviors. Properties of waxy starch gels were mainly governed by amylopectin molecular characteristics, especially in the sugar–acid system. Adding sugar and acid had minor impacts on starch gel behaviors in the linear viscoelastic (LVE) region but were most evident in the nonlinear response regime of starch gels as shown in the Lissajous curves at large oscillatory strain.

## 1. Introduction

Starch, the major carbohydrate in human diets, is composed of two types of glucose polymers: amylose (AM), a linear α−1,4 linked glucan, and amylopectin (AP), a highly branched glucan with α−1,6 glycosidic bonds at branch points. The molecular size of the linear AM is typically ~10–1000 times smaller than the branched AP (DP of AM ~10^2^–10^4^ AGU vs. DP of ~10^4^–10^5^ AGU [1]. Plants synthesize these polysaccharides and store them in insoluble granules or plastids. Granule shape can vary significantly and includes, e.g., spherical, polygonal, and disc-like shapes; granule size can range from 1 μm to more than 100 μm and is determined by botanical origin. The native starch granules of normal starch naturally contain 15–30% AM content whereas waxy starch granules contain < 1% of AM [2]. The molecular arrangement of both glucan polymers within the granule results in a semi-crystalline structure.

The function of starch in food, besides providing an energy source, is to provide structure and texture. Starch is required to undergo gelatinization prior to contributing to any desirable texture. The gelatinization process takes place when heating starch with an excess amount of water (≥65% *w*/*w*). Granules initially swell and AM leaches out. Subsequently, the destruction of crystalline regions in the swollen granules occurs. Thickening, stabilizing, pasting, and gelling are examples of the diversified food properties obtained from gelatinized starch. The AM content, molecular size and shape of the glucan polymers, chemical composition within the granule, and granule characteristics, are the internal factors that control those properties [3].

Previous studies showed the effect of granule size on swelling power (SP) and paste viscosity. Larger granules provided higher SP and viscosity due to their high capacity to hold water [4,5]. Swelling of granules increases their volume fraction, and thus increases starch viscosity. Large swollen granules, therefore, would produce significant viscosity development during initial swelling [6]. Other studies, however, showed contradictory results, and proposed that the high surface area of small granules was more efficient for hydration [7,8].

AP is known to be responsible for granule swelling and the semi-crystalline structure within the granule. Waxy starches usually display greater peak viscosity and higher granular disruption than normal starches, thus providing a sticky paste [3]. In contrast, AM particularly in the presence of lipids inhibits starch swelling, hence providing granule rigidity [9]. A high AM content in starch can result in gels with high storage and loss moduli (G′ and G″), and low loss tangent (Tan δ). These values are indicative of well cross-linked network structures [10,11]. The molecular size and fine structure of AP also affect starch pasting and gelling behaviors. For example, the proportion of long chains in AP was negatively correlated with paste breakdown [12].

Other food ingredients besides the internal factors of starch govern starch gelatinization and starch-based food texture. Sucrose, a common sweetener, increased the gelatinization temperatures and enthalpy of starch [13,14,15]. The authors hypothesized this effect is caused by sugar–water interaction decreasing the amount of available water for starch hydration. Sugar also interacts with starch chains which reduces starch chain flexibility and granular swelling [15]. Renzetti, et al. [16] proposed that the combination of sugar and water can be treated as a “single solvent”; and they argued this single solvent possessed a lower volumetric density of hydrogen bonding sites available in the solution for interaction with starch, compared to water. Hence, their gelatinization temperatures increased. The addition of sucrose concentrations (0–30%) significantly increased the peak viscosity (PV) and final viscosity (FV) of tapioca starch [13]. However, increasing sucrose concentration to ~51% showed a reverse effect by decreasing the PV and FV of wheat starch [17]. The effects of sucrose addition on the rheological properties of starch gels reported in the literature are rather contradictive. The addition of 10–30% sucrose increased storage modulus (G′) of tapioca starch [18]. Ahmad and Williams [19] reported a reduced G′ of sago starch with an increase of sucrose concentrations to 10–40%.

To provide a sour taste, acidic ingredients are sometimes added to a starch-based product, and these can also affect starch gelatinization. Addition of such ingredients can result in starch hydrolysis, altering the starchy texture and its rheological properties. Acid hydrolysis preferentially attacks the amorphous regions since these regions are more accessible due to the loose packing of starch chains compared to the crystalline regions [20]. Chain cleavage of AM and AP mainly occurs at α−1,4 and α−1,6 linkages, respectively [21]. Adding acid solution or pH adjustment to pH ~3 decreased the PV and FV of starch paste [22,23]. Hirashima, et al. [24] also reported a reduced viscosity, and G′ and G˝ moduli of an acidic starch paste, compared to a non-acidified one. They proposed the degradation of granule size and depolymerization of the glucan polymers as possible reasons.

Although the effects of sucrose or acid on pasting and rheological properties of starch have been extensively investigated, limited studies have been done of the concomitant effects of both ingredients on starch behavior, which occur in actual food systems such as fruit-pie filling. Pasting and gelling properties of starches are governed by granule size, AM content, and molecular characteristics of the polymers as previously mentioned. Therefore, the objectives of our study were to investigate the impact of granule size, AM content, and starch molecular characteristics on physico-chemical and rheological properties of starch paste/gels in a neutral system (distilled water) and a sugar–acid system. Normal rice starch, waxy rice starch, normal tapioca starch, and waxy tapioca starch were used in the study, representing small-granule starch and intermediate-granule starch, respectively. To imitate the fruit-pie filling system, the starch concentration was 6% *w*/*v* and the sucrose concentration was 35% *w*/*v*. The pH of the system was adjusted to ~3 using citrate buffer. From an application point of view, the results of this study can provide guidance for use of suitable native starches for desirable texture in clean-label food products e.g., gravies and fruit-pie filling.

## 2. Materials and Methods

### 2.1. Materials

Native tapioca starch (TS) and native waxy tapioca starch (WTS) were obtained from Siam Modified Starch Co., Ltd., Pathum Thani, Thailand. High amylose rice grain—Chainat (CN) and waxy rice grain—Sew Mae Jan (SMJ) were provided by the Rice Department, Thailand. The other ingredients employed were citric acid (Merck, Darmstadt, Germany), sodium citrate (Ajax Finechem, New South Wales, Australia), sucrose (Food grade, Mitr Phol, Bangkok, Thailand) and amylose from potato (Sigma-Aldrich, St. Louis, MO, USA).

### 2.2. Starch Isolation

Rice starches were isolated from two types of rice—CN and SMJ—according to the alkaline method of Lumdubwong and Seib [25] with a slight modification. The starch was extracted using rice grain and wet milling by stone mill; the soaking time was increased to 24 h at 25 °C.

### 2.3. Starch Composition

Moisture content of starch was determined following AACC [26]. Proximate analyses (protein, lipid and ash) of starch were determined following AOAC [27].

Apparent amylose content of starch was measured by the method of Chrastil [28].

### 2.4. Food Model Systems

Native starches were studied in two systems: a neutral system (distilled water), and a sugar–acid system (35% *w*/*v* sucrose and pH 3). The sugar–acid system was adapted from the fruit filling model of Agudelo, et al. [29]. The sugar–acid solution was prepared by adding sucrose in distilled water, followed by continuous stirring for 1 h at room temperature, and adjusting the pH to pH 3 by adding citrate buffer.

### 2.5. Molecular Weight Profile of Native Starches

The molecular weight profiles of the starches were analyzed using high-performance size-exclusion chromatography (HPSEC) with refractive index (RI) detector (UFLC, Shimadzu, Kyoto, Japan). The method was according to Nguyen Vu and Lumdubwong [30]. Six pullulan standards (1 mg/mL) (Showa Denko, Tokyo, Japan) were used to create a standard curve. Weight–average molecular weight ($\bar{M_{w}})$ and number–average molecular weight ($\bar{M_{n}}$) were calculated based on the standard curve. The weight–average degree of polymerization ($\bar{{\mathrm{DP}}_{w}}$) was obtained by dividing $\bar{M_{w}}$ by 162. The amylose content of starch was calculated using the ratio of the peak area of amylose and amylopectin.

The maximum absorbance (λ_max_) of starch was determined by the modified method of Chrastil [28]: 30 mg starch was dissolved in 2 M NaOH (1 mL) and distilled water (2 mL); 100 µL of the starch solution was mixed with 5% TCA (5 ml) and 0.01 N I_2_-KI solution (I_2_ 0.127 g and KI 0.3 mg/ 100 mL) (200 µL). The sample was analyzed by spectrophotometer (GENESYS-10S, Thermo Scientific, Waltham, MA, USA) with a wavelength ranging between 400–900 nm.

### 2.6. Starch Granule Size Distribution and Morphology

The granule size distribution of native starch was determined using a laser particle size analyzer (Mastersizer 2000 with Hydro2000SM, Malvern, Worcestershire, UK). A small amount of native starch was suspended in distilled water. The starch solution was added to circulating water and stirred at 1000 rpm to obtain the particle size. A refractive index (RI) of starch and water of 1.53 and 1.33, respectively, were used for the measurement. The particle size was reported as the median diameter (D_50_). The starch granules’ morphology was examined under a light microscope (Axio-Lab.A1, Carl Zeiss, Oberkochen, Germany) with Micro Publisher-3.3-RTV digital camera.

### 2.7. Measurements of Starch Physico-Chemical Properties

The gelatinization thermal properties of starch were determined by differential scanning calorimetry (DSC, model DSC 1, Stare System, Mettler-Toledo, Greifensee, Switzerland). The ratio of starch: distilled water or sugar–acid solution was 1:2.5. The heating condition was according to Hsieh, et al. [31].

The swelling power (SP) (1% *w*/*v*) of starch in distilled water or sugar–acid solution was determined by the method of Crosbie [32]. The SP of starch was calculated as the weight of sedimented gel, divided by the dry weight of starch minus soluble dry matter. From the SP of starch in sugar–acid solution, no correction for solubles was analyzed because of the high sugar concentrations utilized.

Starch paste clarity was determined by measuring light transmittance (%T). Light transmittance of 1% *w*/*v* starch pastes in distilled water or sugar–acid solution was determined following Craig, et al. [33].

### 2.8. Pasting Properties

The pasting properties of 6% *w*/*v* starch in distilled water or sugar–acid solution were determined by a rapid visco analyzer (RVA, model 4500, Perten Instruments, Stockholm, Sweden). The pasting condition of Lumdubwong and Seib [25] was applied in this study.

### 2.9. Rheological Properties of Starch Gels

Rheological properties of starch gels were analyzed using a rheometer (Model MCR 302, Anton Paar, Graz, Austria) with a stainless-steel parallel plate geometry (50 mm diameter), and gap size of 1.0 mm for all experiments.

#### 2.9.1. Starch Gels Preparation

The 6% *w*/*v* starch slurry in distilled water or sugar–acid solution was pre-heated in a water bath to 60 °C (neutral system) or 70 °C (sugar–acid system) to reduce starch sedimentation. The pre-heated starch slurry was immediately poured onto the lower plate of the Rheometer with the temperature preheated at 50 °C. After lowering the upper plate, a thin layer of silicone oil was applied on the edge of the sample, and a solvent trap was placed on the stainless-steel geometry, in order to prevent moisture evaporation. The slurry was pre-sheared at 10/s for 1 min before starting the test.

All gelation measurements were performed with constant shear strain at 0.5% (which was tested to be in the linear regime) and constant frequency of 1 Hz. The slurry was held at 50 °C for 2 min, then heated up to 95 °C at a rate of 7.5 °C/min, and held at 95 °C for 2 min. Then the starch paste was cooled to 50 °C at 7.5 °C/min, and then held at 50 °C for 4 min. After that the sample was cooled to 25 °C at 7.5 °C/min, and held at 25 °C for 5 min. These starch gels were used in further analysis of small amplitude oscillatory shear (SAOS) and large amplitude oscillatory shear (LAOS) tests.

#### 2.9.2. Small Amplitude Oscillatory Shear (SAOS)

An amplitude sweep test was performed at 25 °C with a shear strain (*γ*) range of 0.01% to 100% (logarithmic ramp mode) and a constant frequency of 1 Hz to determine the extent of the LVE region of the starch gels. For the frequency sweep tests, starch gels prepared in the rheometer (as described in 2.9.1) were allowed to relax for 5 min at 25 °C before starting the test. A frequency range of 0.01 Hz to 20 Hz was used at fixed shear strain within the LVE regime.

#### 2.9.3. Large Amplitude Oscillatory Shear (LAOS)

After the frequency sweep test, the sample was allowed to relax for 10 min at 25 °C before starting the LAOS test. The amplitude was varied in the range of 0.1–1000.0% (logarithmic ramp mode) with constant frequency of 1 Hz, at 25 °C. The responses of the starches from the LAOS tests were analyzed using Lissajous plots and by calculating the dissipation ratio as a function of applied strain amplitude [34,35].

### 2.10. Starch Paste/Gel Microstructure

150 mg of cooked starch paste in distilled water or sugar–acid solution from the study of pasting properties was cooled to room temperature, then stained with 100 μL of iodine solution (1 mL solution containing: 1.67 mg I_2_ and 3.33 mg KI) and analyzed under a light microscope (Axio-Lab.A1, Carl Zeiss, Oberkochen, Germany) with Micro Publisher-3.3-RTV digital camera.

For low-shear preparation, a 6% *w*/*v* starch slurry in distilled water or sugar–acid solution was heated in a water bath (95 °C) for 20 min and periodically stirred gently by hand to reduce starch sedimentation, then cooled to room temperature. The starch gels were stained with iodine as described above and analyzed under a light microscope (Zeiss Axioskop 2 Plus, Oberkochen, Germany, with AxioCam ERc 5S).

### 2.11. Statistical analyses

All measurements were done at least in duplicate. Analysis of variance (ANOVA) and Duncan’s multiple comparison were performed using SPSS 25 software.

## 3. Results and Discussion

### 3.1. Granule Size and Morphology of Native Starches

**Figure 1:**
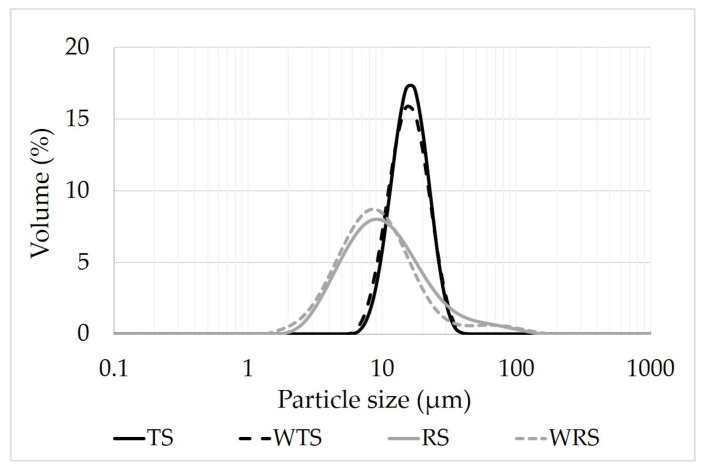
Particle size distribution of native starches.

**Figure 2:**
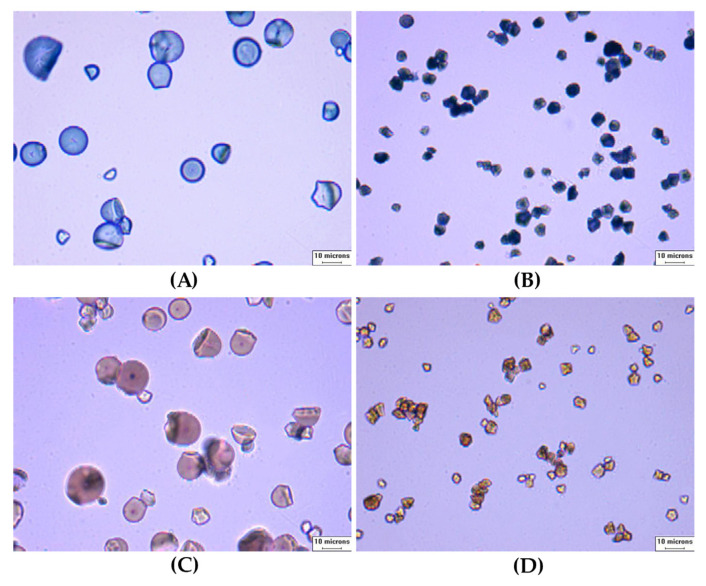
Starch granules morphology (40×, bar = 10 µm): (**A**) TS; (**B**) RS; (**C**) WTS; and (**D**) WRS.

The particle size distribution of rice and tapioca starches is shown in Figure 1. The median diameter (D_50_) of rice starch (~9 μm) and tapioca starch (~15 μm) revealed that their granule size was small (2–10 μm) and intermediate (10–60 μm), respectively [36]. Both starches displayed a unimodal distribution indicating that no irregular aggregation of starches occurred when dispersed in water. Rice starch exhibited a wider particle size distribution, compared to tapioca starch. The presence of compound granules and the angular granule shape of rice were suspected to be the cause of this. The granule size of normal rice starch (RS) was slightly but significantly larger than waxy rice starch (WRS) (9.10 μm vs. 8.19 μm) (*p* < 0.05). RS and WRS granules had angular and polygonal shapes and their granule size was similar (~5 μm) when analyzed by a light microscope (Figure 2B,D). In other studies, the granule size of WRS obtained by SEM was slightly larger than RS granules [37]. The granule size of tapioca starch (TS) was also slightly larger than waxy tapioca starch (WTS) determined by the light scattering method (15.00 μm vs. 14.65 μm) (*p* < 0.05). The results were in agreement with those of Gomand, et al. [38]. The granule shape of TS and WTS was round and truncated and the granule size of both tapioca starches was around ~12 μm when observed under a light microscope (Figure 2A,C). The results show the average granule size of starches was affected by analytical methods.

### 3.2. Chemical Compositions and Molecular Characteristics of Native Starches

The protein, fat, and ash contents of all starches were lower than 0.5%, implying that the starches were pure (Table 1). The higher protein residue in RS compared to WRS is likely related to the granule-bound starch synthase (GBSS), the enzyme responsible for amylose synthesis [39]. The protein residue of TS and WTS was found to be about 0.2%, and their content was similar (*p* ≥ 0.05) independent of their amylose (AM) content. The fat content of starches was as expected since cereal starch has a higher fat content than root/tuber starch.

The RS used in the study was classified as high amylose (HAM) rice, based on its AM content (Table. 1). The overestimation of its AM content determined by the colorimetric method was likely due to an interference of long-chain amylopectin (AP) [40]. The high λ_max_ of RS verified the presence of the linear polymers. TS contained 25% AM which was significantly lower than RS. The λ_max_ and AM content from the iodine-binding method indicated the TS starch also contained long-chain AP. Both waxy starches, WRS and WTS, did not contain small linear molecules of AM according to their molecular distribution (Table 2). The molecular distribution of normal starches (RS and TS) and waxy starches (WRS and WTS) was trimodal and bimodal, respectively (Table 2). The first two fractions were high-molecular weight (HW) AP and low-molecular weight (LW) AP, while the third fraction was AM when classified using the molecular size criteria. The weight-average molecular weight ($\bar{M_{w}}$) of the first fraction of all samples was similar (*p* ≥ 0.05). The data suggested that the molecular size of HW AP was not directly correlated with granule size or amylose content. Nonetheless, waxy starches had a higher proportion of HW AP than normal starches. The molecular size of LW AP of TS was found to be significantly smaller than that of other starches, and WRS contained the highest proportion of LW AP. Different ratios of HW AP to LW AP indicated different heterogeneity of AP polymers which, in turn, affected the short-range and long-range orders of AP arrangement in starch granules. Seemingly, the shape of waxy AP of WTS and WRS was different. The branch points of WTS tended to be closer to the reducing ends compared to those of WRS, based on its higher λ_max_ value (539.8 nm vs. 524.0 nm) as shown in Table 1.

The molecular size of TS AM was about two times larger than that of RS (19.40 × 10^5^ g/mol vs. 7.62 × 10^5^ g/mol). When converted to the weight-average degree of polymerization ($\bar{{\mathrm{DP}}_{w}}$), our findings agreed with a previous study by Breuninger, et al. [41], where the ${\mathrm{DP}}_{w}$ of TS and RS ranged from 580–22,400 AGU and 210–12,900 AGU, respectively

### 3.3. Effect of Granule and Starch Polymer Characteristics, on Physico-Chemical and Pasting Properties

#### 3.3.1. Gelatinization Properties

**Figure 3:**
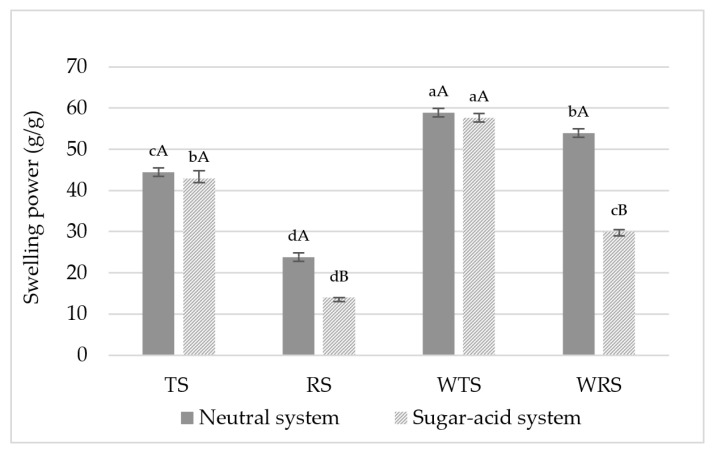
Swelling power (SP) of native starches. The different lowercase letters represented a significant difference (*p* < 0.05) in values compared between types of starch in the same system. The different uppercase letters represented a significant difference (*p* < 0.05) in values compared between systems.

The granule size and AM content evidently influenced the gelatinization properties and swelling power (SP) of starches (Table 3 and Figure 3). The gelatinization properties of starches in the neutral system are shown in Table 3. RS, the AM-containing starch with small granules, showed higher gelatinization temperatures (T_o_, T_p_, and T_c_) than its waxy counterpart (WRS) and those of intermediate-granule starches (TS and WTS) (Table 3). It is known that AM restricts granule swelling, and amylose-lipid complex (ALC) formation can increase gelatinization temperatures [42]. The second endotherm of RS at ~100 °C, most likely an AM–lipid complex, possibly impeded starch hydration, delaying starch gelatinization [42]. The absence of this endotherm of other starches was due to the absence of AM in waxy starches and the very low fat content of normal tapioca starch [41]. Besides AM content and granule size, molecular characteristics also influenced the gelatinization properties of starches. WTS exhibited higher gelatinization temperatures and enthalpy than TS. The difference in chemical composition of the starch granules, molecular size, and shape of the starch polymers were the possible underlying reasons. The average chain length ($\bar{\mathrm{CL}}$) of WTS AP was reported to be slightly longer than TS AP ($\bar{\mathrm{CL}}$_WTS_ 19.5 vs. $\bar{\mathrm{CL}}$_TS_ 19.3) [43]. WTS also exhibited higher proportions of DP 25–DP 36 and DP > 37 than TS [43]. The exceptional, rather long chain length of WTS AP and their external long chain (ECL), as evident from its λ_max_ (Table 1), resulted in a high amount of stable double helical structures and more organized internal structures. As a result, their gelatinization properties were higher than those of AM-containing TS counterparts. The unusually low ΔH of WRS was possibly related to the large heterogeneity of AP molecules previously shown (Table 2). WRS also contained a rather high proportion of short-chain AP (DP 6–12), which could not form stable double helical structures [7,44]. WRS, therefore, contained a lower amount of short-range molecular order than WTS. Thus, less energy was required to disassociate the molecular order. This assumption was confirmed by FTIR measurements (Table S1).

The presence of sugar and acid apparently impacted starch gelatinization properties (Table 3). The T_p_ of all starches in the 35% sucrose-acid system increased ~11–12 °C from that of the neutral system, and was about the same as that of wheat starch in a 30% sucrose solution [16]. Increasing T_p_ of starches in the sugar–acid system was expected to be mainly caused by sucrose. This observation was attributed to two possible reasons. The hydrogen bond interaction between starch and sucrose reduces starch chain flexibility and granule swelling, thus delaying water accessibility into starch granules [15]. Further, the available H-bonding sites for the interaction between starch and the “single solvent” of sugar and water were reduced due to the lower volumetric density of its bonding sites [16]. Our study did not find that citric acid decreased the gelatinization properties when the system was adjusted to pH ~3 and subsequently gelatinized. The results confirm that citric acid, a tricarboxylic acid, did not reduce gelatinization properties, in agreement with the findings of Majzoobi, et al. [45]. Sucrose and acid did appear to affect the AM–lipid complex. The onset melting temperature (T_o_) and the ∆H of the second endotherm of RS increased by 12.7 °C and 0.3 J/g, compared to the neutral system. In contrast, its range of T_c_ − T_o_ was narrowed by ~9 °C (*p* < 0.05). The reason for this observation is most likely that sucrose predominantly interacted with rice AM, yielding granule swelling restrictions, which impeded accessibility to acid hydrolysis.

#### 3.3.2. Swelling Behavior and Paste Clarity

Previously reported effects of granule size on swelling power were contradictory. Cornejo-Ramírez, Martínez-Cruz, Del Toro-Sánchez, Wong-Corral, Borboa-Flores and Cinco-Moroyoqui [7] showed that small starch granules had higher swelling power (SP) than large granules. The observation was explained by the larger surface area of small granules. Sun and Yoo [4], on the contrary, reported that TS displayed higher SP than RS. The authors rationalized their result with the argument that larger granules possessed a higher capacity of water holding. The results of our study were in accordance with those of Sun and Yoo [4]. Swelling power (SP) of intermediate-granule starches, TS and WTS was ~1.5 times that of small-granule starches, RS and WRS (Figure 3). The SP of normal starches was lower than waxy starches, as AM restricts granule swelling as well as provides granule rigidity [9]. RS displayed the lowest SP among all samples. The presence of AM–lipid complex in RS could be an additional factor suppressing granule swelling [9]. Reduced SP of starches cooked in the sucrose solution was initially expected since the system contained a lower amount of available water. The ability of sugars to reduce starch swelling was reported previously to occur when the sugar concentration was higher than 25% [19]. The effect of acid solution on SP was also previously studied; an increase in SP of rice starch was observed when cooked in the 0.2 M acetic acid instead of water. The increase was proposed to be caused by AP molecules absorbing more water in acidic conditions, compared to in water [46]. SP of starches cooked in a solution with sucrose and citric acid are shown in Figure 3. The reduction in SP of RS and WRS is in agreement with the behavior of sago starch cooked in sucrose solution [19]. However, we did not find an effect of acid on the SP of RS and WRS. The SP of tapioca starches in sugar–acid solution was significantly higher than rice starches. The observation suggested that the water diffusion rate through the intermediate granules was relatively higher than the small granules since their water-holding capacity was determined within the same time interval. Further, the water diffusion of tapioca starch granules was also enhanced by absence of AM–lipid complex. We found that the SP of tapioca starches in sugar–acid solution was similar to those in distilled water (*p* ≥ 0.05). The observation suggested that water absorbability of tapioca AP was likely increased by presence of acid resulting in an increase of SP, as described by Ohishi, Kasai, Shimada and Hatae [46]. This possibly occurred prior to the effect of sucrose, which reduced starch swelling. Hence, the counteracting effects of acid and sugar resulted in a similar SP of tapioca starches in neutral and sugar–acid systems. In contrast, a restriction of granule swelling due to sucrose predominantly occurred in small granules. The SP behavior of rice, therefore, followed the decreasing trend of the SP of cooked starch in sucrose solution compared to water observed in previous studies.

Paste clarity of starches in distilled water (Table 1) was in accordance with their SP except WRS. It has been reported that starch paste clarity was governed by chemical compositions and AM content [33], and the dispersion of starch granules [47]. Rice starches showed significantly lower light transmittance (%T) than tapioca starches (*p* < 0.05). The high AM content of RS granules preserved the integrity of granule remnants; therefore, less light could pass through the paste. WRS also displayed a rather low paste clarity which is similar to the finding of Hsieh, Liu, Whaley and Shi [31]. The reasons why WRS exhibited a rather low %T despite high SP, absence of AM, and low minor components, are still unclear and further studies are required to elucidate this observation. The paste clarity of starches in the sugar–acid system increased, except RS. Increasing paste clarity was in agreement with the study of Craig, Maningat, Seib and Hoseney [33]. The authors reported an increase of starch paste clarity in the presence of sucrose. They suggested that the addition of sucrose increased the refractive index of the solution surrounding swollen granules, hence reducing light refraction. Additionally, sucrose retarded the association of starch chains leading to lower whiteness [33]. The 200% reduction of paste clarity of RS in the sugar–acid system was thought to be due predominantly to sucrose restricting granule swelling.

#### 3.3.3. Pasting Properties of Native Starches

##### Pasting Temperature (PT) and Peak Viscosity (PV)

**Figure 4:**
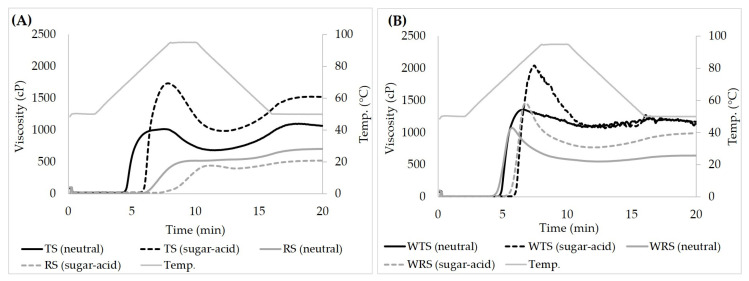
Pasting properties of native starches: (**A**) normal starches and (**B**) waxy starches (starch concentration: 6% *w*/*v*). The solid lines and dashed lines represent starch pasting in distilled water and sugar–acid solution, respectively.

Pasting profiles of normal and waxy starches in water and sugar–acid solution are shown in Figure 4. Pasting temperatures (PT) of all samples in sugar–acid solution shifted up ~10–12 °C from those in distilled water, following the trend of their gelatinization temperatures (T_o_, T_p_, and T_c_) (Table 3). The peak viscosity (PV) is described as the consistency of a starch solution when the maximum granule swelling rate is equal to the granule disruption rate during heating [48]. The PV of starches was consistent with their SP as a positive correlation between PV and SP was reported by Crosbie [32]. The lower PV of TS and RS compared to WTS and WRS is most likely due to AM restricting granule swelling in the former. The higher PV of TS and WTS compared to RS and WRS implied that the larger granule size provided a higher degree of granule expansion. In the presence of sugar and acid, the PV of starches increased by about 40–70% compared to the neutral system, except RS (Figure 4). The increase in PV of starches in sugar–acid solution is similar to the increasing trend of the PV of wheat starch in the sucrose solution [17]. Woodbury, Grush, Allan and Mauer [17] observed an increase in the PV of wheat starch in 1.0 M sucrose solution (2.0 M_mono_ solution). They explained that the granule rigidity during heating and mixing increased due to sucrose. The sugar not only competed with starch for water, but increased H-bonding within the granule. The relative increase in PV in the sugar (1.0 M sucrose)–acid (0.1 M citric acid) system in our study was found to be similar to the relative increase of PV in the 1.0 M sucrose solution by Woodbury, Grush, Allan and Mauer [17]. Seemingly, it was predominantly the sucrose that affected PV and acid hydrolysis did not significantly take place at this stage. It appears that the granule rigidity of RS was extremely high, yielding no expansion in the sugar–acid system and, thus the lowest PV.

##### Breakdown (BD) and Trough Viscosity (T)

The breakdown (BD) viscosity is the difference between PV and trough viscosity (T) or hot paste viscosity (HV). The parameter is associated with the disruption of gelatinized swollen granules. The low BD represents the resistance to shear or granule rigidity before disruption. The trough viscosity is affected by solid content e.g., granule remnants and other ingredients (e.g., sugar and acid), amount and molecular characteristics of solubles. These factors provide the volume fraction and granule rigidity which govern the T. Our results showed intermediate-granule starch had a higher T than small-granule starch. Kowittaya and Lumdubwong [49] observed positive correlations of T and $\bar{CL}$ and $\bar{ECL}$ of rice AP. Therefore, the long $\bar{CL}$ and $\bar{ECL}$ of WTS were likely the causes of its higher T, compared to TS. WRS showed slightly higher T than RS, even though WRS has low granule rigidity. The high disruption of WRS granules possibly resulted in a high concentration of soluble AP which provided viscosity to WRS paste after granular disintegration. The increased T values of all starches except RS prepared in sugar–acid solution was in agreement with the increased T of wheat starch prepared in 2.0 M_mono_ sucrose solution [17]. The relative increase of T for all samples, nonetheless, was lower than reported in that study. It indicates that acid hydrolysis could have occurred and had a negative effect on consistency. The different starches exhibited a different degree of interactions with sucrose and had dissimilar levels of acid hydrolysis. WTS was the most susceptible to acid hydrolysis based on its highest BD and its unchanged T in sugar–acid solution compared to water. The acid susceptibility was considered to be due to the intermediate granule size with no presence of AM since these observations (high BD and unchanged T) were not found in WRS. On the contrary, WRS showed an increase in T and lower BD compared to WTS in the sugar–acid system despite the absence of AM and a low amount of short-range molecular order of WRS (Table 3). The high proportion of long-chain AP of WTS also facilitated acid hydrolysis, as suggested by Kim, et al. [50]. Based on the 50% increase of T and the 100% increase of BD of TS in sugar–acid solution, the ability to interact between sucrose and starch polymers and acid hydrolysis occurred during starch pasting. In contrast, the high AM content, AM–lipid complex formation, and small granule size of RS impeded granule swelling. The favorable interaction between AM and sucrose also reduced AM leaching and restricted granule swelling thus lowering the degree of acid hydrolysis of RS. The explanation was confirmed by the lowest BD of RS in sugar–acid solution compared to other starches.

##### Setback (SB) and Final Viscosity (FV)

**Figure 5:**
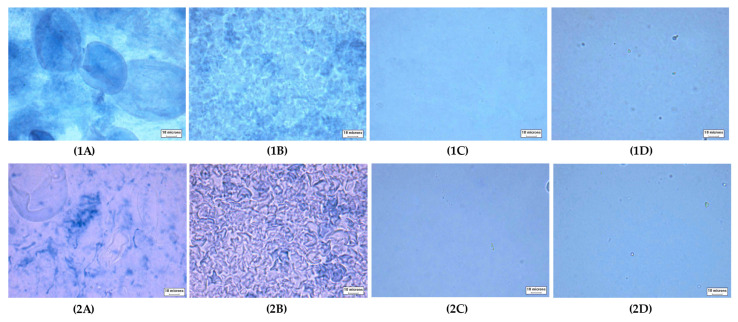
Microstructure of cooked starch pastes (RVA preparation) (40×, bar = 10 µm). Uppercase letters indicate type of starches: (**A**) TS, (**B**) RS, (**C**) WTS, and (**D**) WRS. The number indicate the system used to prepare starch gels; (**1** neutral system and (**2**) sugar–acid system. (**1A**): TS paste cooked in a neutral system; (**1B**): RS paste cooked in a neutral system; (**1C**): WTS paste cooked in a neutral system; (**1D**): WRS paste cooked in a neutral system; (**2A**): TS paste cooked in a sugar-acid system; (**2B**): RS paste cooked in a sugar-acid system; (**2C**): WTS paste cooked in a sugar-acid system; (**2D**): WRS paste cooked in a sugar-acid system.

The setback (SB), the difference between final viscosity (FV) and T, and FV in distilled water are generally regulated by recrystallization of starch polymers (mainly AM), molecular entanglement (gel formation), and the volume fraction of granule remnants. The SB indicates gelling ability and/or retrogradation ability [51]. Thus, low SB suggested low gelling ability and/or a low degree of retrogradation. The high SB of TS and RS is in agreement with their high AM component, and granule remnants of both starches were observed (Figure 5(1A,1B)). The FV of TS was found to be almost double that of RS. The high $\bar{{\mathrm{DP}}_{w}}$ of TS AM (Table 2) reasonably promoted entanglement and recrystallization. Further, the large granule volume of TS increased its FV as described by Waterschoot, et al. [52]. Large swollen granule remnants interacting with the leached materials could reinforce the starch network, thus increasing viscosity during cooling [52]. The SB, the difference between FV and T, of Wx starches was 1.5–8 times lower than normal starches. The absence of AM and very low volume fraction of remnants clearly caused their small SB (Figure 5(1C,1D)). The superior FV of WTS compared to WRS was likely due to its AP molecules with high $\bar{CL}$ and $\bar{ECL}$, and the higher volume fraction of granule remnants. The increased FV of all starches in sugar–acid solution, except RS, followed the increased trend of the FV of starch in sucrose solution [17]. However, the increase in SB of starches in sugar–acid solution was opposite to the previous report where the SB decreased in sucrose solution compared to water [17]. The observation confirmed the occurrence of acid hydrolysis during starch pasting in our study. It was proposed that acid promoted starch granule disruption during the heating stage. The chain-cleavage resulted in leaching out of depolymerized starch polymers [53]. The extent of hydrolysis in waxy starches was very high as shown by the high percentage increase of SB of WTS and WRS (180% and 170%, respectively). The FV of waxy starches was suggested to be mainly resulting from AP polymer gel phase, since both systems displayed a very clear paste with almost no remnants (Figure 5(2C,2D)). Evidence of acid hydrolysis was also found in TS, as indicated by the 50% increase of SB. The highest FV of TS can likely be attributed to the higher amount of leached AM with high DP_w_ and high volume fraction of large swollen granule remnants (Figure 5(2A)). The assumption was confirmed by the increased ratio of the blue color intensity between the soluble phase and swollen granule remnants. This observation suggested an increase of AM leaching. Acid hydrolysis also took place in the soluble phase, and degradation of linear molecules occurred as shown by the shift from the blue background of the iodine-stained paste in water to purple (Figure 5(2A,2B)). RS showed an extremely low FV and a reduced SB in sugar–acid solution compared to water. This observation was attributed to extensive sucrose–starch interaction within granules that restricted granule swelling [15]. This effect resulted in a low-volume fraction of granule remnants and lower extent of AM leaching. Therefore, a low concentration of acid-hydrolyzed AM in the soluble phase was obtained. This assumption was supported by the intense blue color in swollen granule remnants of RS and less blue color intensity in the background compared to the neutral system (Figure 5(2B)).

### 3.4. Effect of Granule and Starch Polymer Characteristics on Rheological Properties of Starch Gels

#### 3.4.1. Small Amplitude Oscillatory Shear (SAOS)

**Figure 6:**
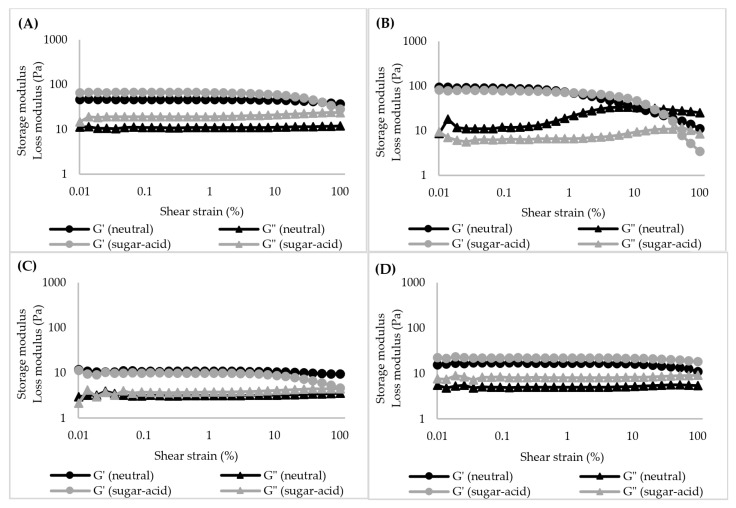
Storage modulus (G′) and loss modulus (G″) of starch gels (6% *w*/*v*) as a function of shear strain for the neutral system and sugar–acid system: (**A**): TS, (**B**): RS, (**C**): WTS, and (**D**): WRS.

**Figure 7:**
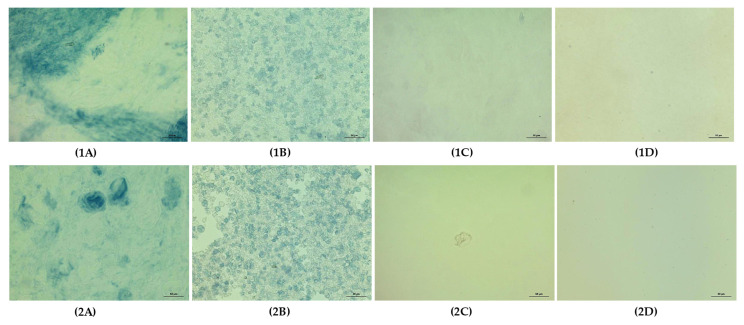
Microstructure of starch gels (Low-shear preparation) (20×, bar = 50 µm). Uppercase letters indicate type of starches: (**A**): TS, (**B**): RS, (**C**): WTS, and (**D**): WRS. The number indicate the system used to prepare starch gels; (**1**): neutral system and (**2**): sugar–acid system. (**1A**): TS gel in a neutral system; (**1B**): RS gel in a neutral system; (**1C**): WTS gel in a neutral system; (**1D**): WRS gel in a neutral system; (**2A**): TS gel in a sugar-acid system; (**2B**): RS gel in a sugar-acid system; (**2C**): WTS gel in a sugar-acid system; (**2D**): WRS gel in a sugar-acid system.

From the SAOS measurements, the extent of the linear viscoelastic (LVE) region and the effect of frequency on G′ and G″ values of starch gels were determined. The extents of the LVE region of starch gels in water (neutral system) or sugar–acid solution (sugar–acid system) are presented in Figure 6. The G′ values of both normal starch gels were significantly higher than those of waxy starch gels (*p* < 0.05). The observation clearly indicates the different characteristics of these starch gels, in addition to the starch paste viscosities from the RVA measurements. This demonstrates the importance of AM that maintains the integrity of swollen granules and strengthens the system by forming polymer networks of leached AM, giving elasticity to starch gels [54]. In contrast, waxy starch consists of highly branched molecules that are typically not able to form gels as strong as those of AM, therefore, their gels are soft and highly viscous, consistent with their high FV (Figure 4B). The small granules with high AM content of RS resulted in a significantly higher G′ value and lower loss tangent (Tan δ) compared to TS gel (*p* < 0.05) (Figure 6A,B). In contrast, both waxy starch gels showed similar G′ and Tanδ values in the LVE region (*p* ≥ 0.05) (Figure 6C,D). The impact of starch granule size is not pronounced on waxy starch gel behaviors since almost no granule remnants remained in their gels (Figure 7(1C,1D)). Further, molecular characteristics also did not evidently affect waxy starch gel properties in this stage. The maximum linear strain (*γ*_max_) of RS was very short, with a value of around 0.2% (Figure 6B), whereas other starch gels showed much longer LVE regions, with *γ*_max_ in the range of ~10–15% (Figure 6A,C,D). Furthermore, RS gel presented a shear-thickening behavior in the G˝ curve, known as weak strain overshoot behavior, which is absent in other starch gels [55]. The results emphasized that high AM content, small granule size, and the presence of AM–lipid complex in RS promoted the rigidity of the gel. This is possibly due to the restricted swelling of RS granules, therefore, remaining as rigid swollen granules in the gel, as is also evident from the intact granule remnants observed in the microscopy images (Figure 7(1B)). In particle-filled gels, the weak strain overshoot is often caused by collision of these particles, leading temporarily to the formation of larger clusters, in turn leading to an increase in G″. After a further increase in shear strain, these clusters are broken down again, and hence G″ decreases, giving rise to a maximum in G″. The results indicated that the RS gel is stronger and more brittle than other starch gels, which are softer and more stretchable. 

**Figure 8:**
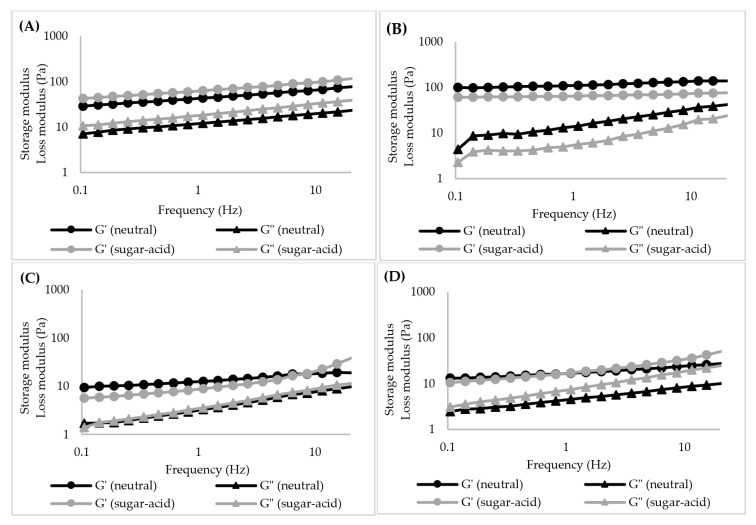
Storage modulus (G′) and loss modulus (G″) of starch gels (6% *w*/*v*) as a function of frequency for the neutral system and sugar–acid system: (**A**): TS, (**B**): RS, (**C**): WTS, and (**D**): WRS.

In the sugar–acid system, the G′ value of TS gel increased compared to that of the neutral system (70 Pa vs. 43 Pa) (*p* < 0.05), whereas other starch gels displayed similar values of G′ to those in the neutral system (*p* ≥ 0.05) (Figure 6). In addition, G″ of the WRS gel increased significantly compared to that of the neutral system (8 Pa vs. 5 Pa) (*p* < 0.05) (Figure 6D), therefore the presence of sugar and acid clearly increases the viscous properties of the WRS gel. Our results show no significant difference in Tanδ values of the starch gels between neutral and sugar–acid systems (*p* ≥ 0.05). However, the extent of the LVE regime of TS and WTS gels became shorter compared to the neutral system (Figure 6A,C) whereas the LVE regime for RS and WRS gels were slightly longer (Figure 6B,D). The G′ and G″ values of starch gels in water and sugar–acid solution as a function of frequency are presented in Figure 8. The frequency range was limited to 0.1 Hz–20 Hz due to the low values for the moduli, which resulted in very noisy results at frequencies below 0.1 Hz, and inertial effects above 20 Hz. The G′ and G″ values of TS, WTS, and WRS gels displayed a gradual increase as the frequency was raised, with a power-law dependence of the moduli on frequency (Figure 8A,C,D). The G′ value of the RS gel was nearly independent of frequency within the tested range (Figure 8B). In addition, the frequency dependence of the starch gels in the sugar–acid system was similar to that in the neutral system.

#### 3.4.2. Large Amplitude Oscillatory Shear (LAOS)

**Figure 9:**
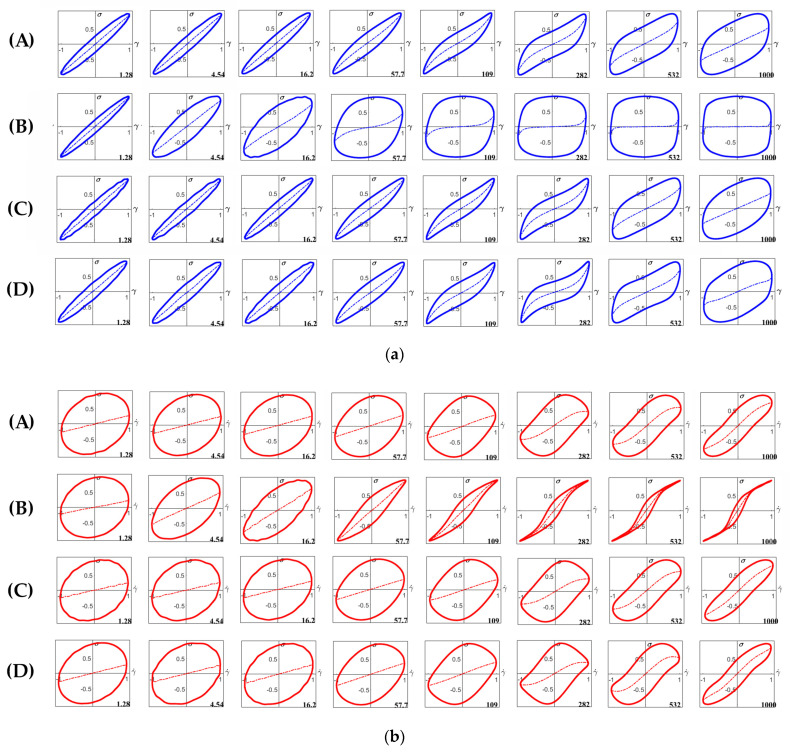
(**a**) Lissajous plots of stress versus strain (τ (*γ*)) for starch gels in the neutral system with shear strains (*γ*) of (from left to right) 1.28%, 4.54%, 16.2%, 57.7%, 109%, 282%, 532% and 1000% at 25 °C: (**A**) TS, (**B**) RS, (**C**) WTS, and (**D**) WRS. Dashed curves inside the loops indicate the contribution of the elastic stress to the total stress [34]. (**b**) Lissajous plots of stress versus strain rate (*τ* ($\dot{\gamma }$)) for starch gels in the neutral system with shear strains (*γ*) of (from left to right) 1.28%, 4.54%, 16.2%, 57.7%, 109%, 282%, 532% and 1000% at 25 °C: (**A**) TS, (**B**) RS, (**C**) WTS, and (**D**) WRS. Dashed curves inside the loops indicate the contribution of the viscous stress to the total stress [34].

**Figure 10:**
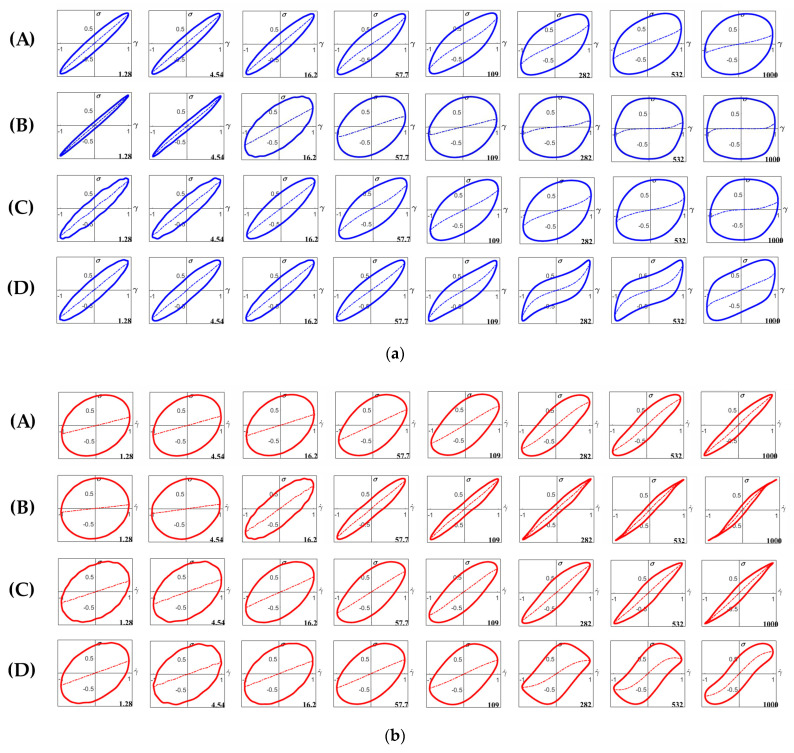
(**a**) Lissajous plots of stress versus strain (τ (*γ*)) for starch gels in the sugar–acid system with shear strains (*γ*) of (from left to right) 1.28%, 4.54%, 16.2%, 57.7%, 109%, 282%, 532% and 1000% at 25 °C: (**A**) TS, (**B**) RS, (**C**) WTS, and (**D**) WRS. Dashed curves inside the loops indicate the contribution of the elastic stress to the total stress [34]. (**b**) Lissajous plots of stress versus strain rate (*τ* ($\dot{\gamma }$)) for starch gels in the sugar–acid system with shear strains (*γ*) of (from left to right) 1.28%, 4.54%, 16.2%, 57.7%, 109%, 282%, 532% and 1000% at 25 °C: (**A**) TS, (**B**) RS, (**C**) WTS, and (**D**) WRS. Dashed curves inside the loops indicate the contribution of the viscous stress to the total stress [34].

**Figure 11:**
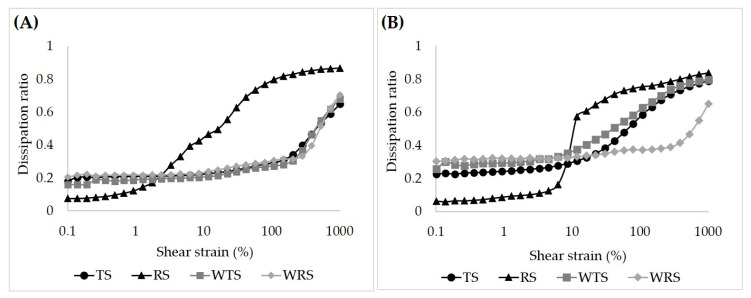
Dissipation ratio of native starch gels (6% *w*/*v*): (**A**) neutral system and (**B**) sugar–acid system.

The behavior of the starch gels beyond the LVE region was determined by LAOS measurements. Lissajous (LJ) and dissipation ratio curves are shown in Figure 9, Figure 10 and Figure 11, respectively. The elastic LJ plots of starch gels in the neutral system (Shear stress-strain plots) are shown in Figure 9a. The curves for TS, WTS, and WRS gels have a narrow elliptical shape at low shear strain, indicating a predominantly elastic response (Figure 9a(A,C,D)). The curves for these gels remain elliptical in shape up to a shear strain (*γ*) of 16% and then start to gradually distort to a rhomboidal shape. RS gels exhibited markedly different characteristics compared to the other starch gels. At strains of 4.5% and 16%, the RS gel still showed a near elliptical shape, but the loops were much wider, indicating a significantly higher viscous dissipation. This was followed by a rapid transition to a wide rhomboidal shape at 58% strain (Figure 9a(B)). At 1000%, the curve of the RS gel displayed an almost square shape with a notably high dissipation ratio (0.87), indicating a near plastic behavior and a high degree of disruption of gel structure (Figure 11A). In contrast, at the highest shear strain (1000%), the TS, WTS and WRS gels retained a significant level of elasticity, as evident from the finite slope of the dashed curve within the LJ plots, which indicates the contribution of the elastic stress to the total stress [34]. At a strain of 282%, TS, WTS, and WRS, even show a small upswing in the elastic stress towards maximum strain, indicating a mild degree of strain hardening. This effect is strongest for the WRS gel. The dissipation ratios were also significantly lower, around 0.62–0.72 (Figure 11A). The result confirms our previous assumption that the RS gel is stiffer, and more brittle compared to the other starch gels that are more stretchable.

The viscous plots of starch gels in the neutral system (shear stress-strain rate LJ plots) are presented in Figure 9b. The curves for TS, WTS, and WRS starch gels showed gradual transitions from elliptical shape to a wide, slightly sigmoidal shape (Figure 9b(A,C,D)). In contrast, the RS gel displayed a more abrupt change from an elliptical shape to a narrow sigmoidal shape, indicating a predominantly viscous response with strong shear-thinning behavior (Figure 9b(B)). 

The elastic plots of starch gels in the sugar–acid system are shown in Figure 10a. At 1–5% strain, the curves for RS showed very narrow elliptical shapes, indicating a predominantly elastic response with high stiffness (Figure 10a(B)); in comparison, the loops in the neutral system were significantly wider. However, in the sugar–acid system, the transition to plastic behavior was more abrupt for RS, as shown by the sudden increase of dissipation ratio at shear strain beyond ~6% (Figure 11B). Interestingly, for TS and WTS gels the transition to rhomboidal-shaped plots shifted to lower strains and the plots became notably wider, particularly at 1000% strain, compared to the neutral system (Figure 10a(A,C)). This observation suggested that their gel structures were more disrupted, as evident by an increase in the dissipation ratio values at 1000% (Figure 11B). In contrast, the curves for WRS gel showed only small changes from the neutral system (Figure 10a(D)). 

The viscous plots of starch gels in the sugar–acid system are shown in Figure 10b. The curves for the RS gel again displayed a change from a wide elliptical shape to a narrow loop, but the sigmoidal shape was less pronounced than in the neutral system, indicating less shear-thinning occurred at high strain rate (Figure 10b(B)). The curves for TS and WTS also were narrower than in the neutral system, indicating a relative increase of the viscous contribution to the total stress (Figure 10b(A,C)). The curve for the WRS gel remained similar in behavior to those for the neutral system (Figure 10b(D)). 

Clearly, the addition of sugar and acid affects the nonlinear response of the starch gels. Regarding normal starches, sucrose inhibits starch swelling and AM leaching, as evidenced by an increase of dark-blue stains within their swollen granules (Figure 7(2A,2B)). The effect of sucrose promotes the rigidity of swollen granules of TS and the system appears to have more ghost remnants (Figure 7(2A)). This has only a minor effect on stiffness but does shift the maximum linear strain to lower values. These observations suggest that the TS gel starts to behave more like a particle gel rather than a polymer gel. For RS, which in the neutral system already behaves more like a particle (-filled) gel, decreased granule swelling apparently decreased the volume fraction of granule remnants (Figure 7(2B)). This resulted in a significant decrease in the weak strain overshoot in G”, and a slightly longer LVE region of the RS gel compared to the neutral system (Figure 6B). The observations suggest that the effect of acid was less pronounced in these samples. Differences in swelling behavior, granule rigidity, volume fraction of particles, and the amount of leached-out AM, appear to be the main factors influencing normal starch gel properties. 

In both waxy starch gels, almost no granule remnants were observed, implying that acid promotes granular disruption even in a low-shear preparation, and their gels are polymer networks of AP (Figure 7(2C,2D)). The different gel behaviors of the waxy starches in the sugar–acid system must be caused by the characteristics of the AP molecules and the chains in the system after chain-cleavage by the acid. The high proportion of long-chain AP of WTS may facilitate acid hydrolysis in amorphous regions [50]. This may have led to a system with more but shorter chains with increased linearity, and this had a shorter LVE regime and a relatively more viscous response in the nonlinear regime. Partial recrystallization of the shorter more linear molecules could possibly be a cause for a more brittle gel structure being formed, and the notably shorter LVE region range and the increase in dissipation ratio observed in the WTS gel (Figure 6C and Figure 11B). Seemingly, acid hydrolysis occurs less in the WRS gel. The increase in G” value and the longer LVE region range of the WRS gel compared to in the neutral system likely indicate that WRS AP molecules remains highly branched, which provides high viscosity to the system. Accordingly, differences in molecular characteristics and AP fine structure were possible reasons that governed the extent of acid hydrolysis and affected waxy starch gel behaviors.

The rheological measurements revealed different behaviors of starch gels. RS gel was the strongest and the most brittle gel in both neutral and sugar–acid systems, compared to other starch gels which are softer and quite stretchable. Further, the results of the LAOS measurements indicated that WRS produced soft and the least brittle gel among other starches in the sugar–acid system. In addition, WRS paste was not extremely turbid as its paste clarity increased to ~55% (%T) in the sugar–acid system (Table 1). The differences between pasting parameters (PV, T, and FV) of WRS were also rather small in both systems (Figure 4), which in turn facilitated the shear-mixing process. The results suggest that WRS is a good selection for fruit-pie filling and gravy sauce. Note here that the cooking temperature of these starches in the presence of sugar and citric acid ingredients is higher by approximately 10 °C than in the neutral system, as shown by their gelatinization temperatures (Table 3).

## 4. Conclusions

Intermediate-granule starches displayed higher SP and volume fraction, compared to small-granule starches in both neutral and sugar–acid systems. The synergistic effect between small granule size and the presence of AM drastically restricted granule swelling and AM leaching in the sugar–acid system. The observation was confirmed by the lowest PV and the microscopy images of RS paste. Gelatinized starches with intermediate granule size were susceptible to acid hydrolysis and sucrose effect, as shown by their higher BD and SB than the small-granule starches. In contrast, sucrose effect was more prominent in small-granule starch, containing AM. The high molecular weight ($\bar{M_{w}}$) and linearity of AP and AM molecules increased the consistency of starch pastes in both neutral and sugar–acid systems. However, granule size displayed a stronger impact on starch paste consistency than the presence of AM and starch molecular profiles, particularly in the sugar–acid system.

The rheological behavior of starch gels was investigated by SAOS and LAOS measurements. The granule size and AM content showed an interaction effect on normal starch gel behaviors in both neutral and sugar–acid systems. RS produced a stronger and more brittle gel than other starch gels in both systems due to its small granule size, high AM content, and the presence of AM–lipid complex. The impact of starch granule size, however, was not pronounced on waxy starch gel behaviors, since their gels are AP polymer networks. In turn, the waxy starch gel behaviors were governed by AP molecular characteristics, especially in the sugar–acid system. It was found that the addition of sugar and acid showed minor impacts on starch gel behaviors in the LVE region but more evidently affected the nonlinear response of starch gels, as shown in the Lissajous curves.

## Figures and Tables

**Table 1:** Chemical compositions, amylose content, maximum wavelength, and light transmittance of native starches.

Sample	Chemical Compositions			Amylose Content (%)		Maximum Wavelength (nm)	% Light Transmittance (%T)	
	Protein (%)	Fat (%)	Ash (%)	Colorimetric Method	HPSEC Method		Neutral System	Sugar–Acid System
TS	0.23 ±0.03 ^b^	0.06 ±0.01 ^ab^	0.23±0.01 ^a^	25.92 ±0.78 ^b^	21.71 ±1.13 ^b^	581.0 ± 3.6 ^a^	56.2 ± 0.1 ^b^	72.2 ± 0.1 ^b^
WTS	0.22 ±0.01 ^b^	0.01 ±0.01 ^b^	0.18 ±0.01 ^a^	0.39 ±0.55 ^c^	0.00 ±0.00 ^c^	539.8 ± 2.9 ^b^	62.1 ± 1.0 ^a^	85.3 ± 0.1 ^a^
RS	0.47 ±0.03 ^a^	0.08 ±0.03 ^a^	0.21 ±0.03 ^a^	36.89 ±0.94 ^a^	30.91 ±0.26 ^a^	581.8 ± 3.0 ^a^	13.9 ± 0.1 ^d^	6.5 ± 0.1 ^d^
WRS	0.22 ±0.10 ^b^	0.13 ±0.03 ^a^	0.16 ±0.02 ^a^	0.28 ±0.39 ^c^	0.00 ±0.00 ^c^	524.0 ± 0.0 ^c^	18.6 ± 0.5 ^c^	54.5 ± 0.1 ^c^

Mean values in the same column with different letters are significantly different (*p* < 0.05).

**Table 2:** Molecular distribution and AM/IM/AP ratio of starches.

Sample	Fraction I (AP)				Fraction II (IM)				Fraction III (AM)			
	$\bar{M_{w}}$ (×10^7^ g/mol)	$\bar{M_{n}}$ (×10^7^ g/mol)	$\bar{DP_{w}}$ (×10^5^)	%Area	$\bar{M_{w}}$ (×10^6^ g/mol)	$\bar{M_{n}}$ (×10^6^ g/mol)	$\bar{DP_{w}}$ (×10^4^)	%Area	$\bar{M_{w}}$ (×10^5^ g/mol)	$\bar{M_{n}}$ (×10^5^ g/mol)	$\bar{DP_{w}}$ (×10^3^)	%Area
TS	6.75 ^a^	5.17 ^a^	4.17 ^a^	66.31 ^c^	6.81 ^b^	5.50 ^b^	4.20 ^b^	11.98 ^b^	19.40 ^a^	16.20 ^a^	12.00 ^a^	21.71 ^b^
WTS	6.52 ^a^	5.00 ^a^	4.02 ^a^	87.25 ^a^	13.90 ^a^	11.10 ^a^	8.60 ^a^	12.75 ^b^	nd	nd	nd	nd
RS	6.70 ^a^	5.14 ^a^	4.14 ^a^	52.76 ^d^	12.20 ^a^	9.76 ^a^	7.55 ^a^	16.54 ^b^	7.62 ^b^	6.48 ^b^	4.71 ^b^	30.91 ^a^
WRS	6.17 ^a^	4.74 ^a^	3.81 ^a^	75.55 ^b^	11.50 ^a^	9.19 ^a^	7.10 ^a^	24.46 ^a^	nd	nd	nd	nd

Mean values in the same column with different letters are significantly different (*p* < 0.05). AP = amylopectin; IM = intermediate materials; AM = amylose.

**Table 3:** Gelatinization properties of native starches.

Sample	Peak I					Peak II				
	T_o_ (°C)	T_p_ (°C)	T_c_ (°C)	T_c_ − T_o_ (°C)	∆H (J/g)	T_o_ (°C)	T_p_ (°C)	T_c_ (°C)	T_c_ − T_o_ (°C)	∆H (J/g)
Neutral system										
TS	64.00 ± 0.21 ^c^	70.75 ± 0.11 ^c^	82.25 ± 0.17 ^c^	18.26 ± 0.04 ^a^	10.62 ± 0.72 ^b^					
WTS	68.78 ± 0.09 ^b^	74.42 ± 0.12 ^b^	84.96 ± 0.30 ^b^	16.18 ± 0.21 ^b^	12.63 ± 0.41 ^a^					
RS	76.51 ± 0.06 ^a^	81.42 ± 0.12 ^a^	89.06 ± 0.09 ^a^	12.55 ± 0.03 ^c^	11.19 ± 0.31 ^b^	95.96 ± 0.00	107.83 ± 0.00	117.25 ± 0.30	21.29 ± 0.30	0.08 ± 0.04
WRS	63.88 ± 0.38 ^c^	70.92 ± 0.35 ^c^	82.30 ± 0.59 ^c^	18.42 ± 0.21 ^a^	10.69 ± 0.45 ^b^					
Sugar-acid system										
TS	75.28 ±0.02 ^c^	82.59 ±0.12 ^d^	95.52 ±0.21 ^b^	20.25 ±0.23 ^a^	13.19 ±0.71 ^a^					
WTS	79.85 ±0.04 ^b^	85.50 ±0.00 ^b^	99.87 ±0.72 ^a^	20.02 ±0.76 ^a^	13.94 ±0.21 ^a^					
RS	88.46 ±0.08 ^a^	93.09 ± 0.12 ^a^	100.71 ±0.17 ^a^	12.25 ±0.08 ^b^	11.15 ±0.71 ^b^	108.64 ± 0.40	115.50 ± 0.24	121.04 ± 0.2	12.40 ± 0.18	0.39 ± 0.04
WRS	74.96 ±0.07 ^d^	82.92 ±0.12 ^c^	95.08 ±0.71 ^b^	20.12 ±0.64 ^a^	13.15 ±0.88 ^a^					

Mean values in the same column with different letters are significantly different (*p* < 0.05). T_o_ = onset temperature of gelatinization; T_p_ = peak temperature; T_c_ = conclusion temperature, ∆H = gelatinization enthalpy.

## Data Availability

Not applicable.

## Supplementary Materials

The following supporting information can be downloaded at: https://www.mdpi.com/article/10.3390/foods11244060/s1, Table S1: The ratio of the 1047 cm^−1^/1022 cm^−1^ absorbance heights (Crystalline/amorphous regions).
