# Supplementary material for: Pasting and Rheological Properties of Starch Paste/Gels in a Sugar-Acid System
Source: Foods. 2022 Dec 15;11(24):4060. doi: 10.3390/foods11244060 (PMC9778545; doi:10.3390/foods11244060)
Supplement: Supplementary file 1 [file foods-11-04060-s001.zip › foods-2040170-supplementary.pdf]

Supplementary Materials

Table S1. The ratio of the 1047 cm^{-1}/1022 cm^{-1} absorbance heights (Crystalline/amorphous regions)

**Sample**	**Ratio of absorbance heights** (1047 cm^{-1}/1022 cm^{-1})
TS	0.67 ± 0.01^{a}
WTS	0.68 ± 0.01^{a}
RS	0.65 ± 0.01^{b}
WRS	0.58 ± 0.00^{c}

*Mean values in the same column with different letters are significantly different ($p < 0.05$).

* TS: tapioca starch, WTS: waxy tapioca starch, RS: rice starch, and WRS: waxy rice starch.
